# Supplementary material for: Effects of Thiamethoxam-Dressed Oilseed Rape Seeds and Nosema ceranae on Colonies of Apis mellifera iberiensis, L. under Field Conditions of Central Spain. Is Hormesis Playing a Role?
Source: Insects. 2022 Apr 9;13(4):371. doi: 10.3390/insects13040371 (PMC9032297; doi:10.3390/insects13040371)
Supplement: Supplementary file 1 [file insects-13-00371-s001.zip › insects-1662776-supplementary.pdf]

Supplementary information

Table S1 Soil characteristics of experimental plots (mean± sd, n= 3).

**Parameters**	**Control plot**	**TMX plot**
Date of sampling	October 2014	October 2014
Sample depth (cm)	0-20	0-20
Code.	Cerro	Pinos
USDA texture classification	Clay	Clay loam
% sand (>0.05 mm)	31.350 ± 1.250	37.600 ± 2.500
% silt (0.05-0.002 mm)	25.000 ± 2.887	26.250 ± 2.394
% clay (< 0.002 mm)	43.650 ± 3.750	36.150 ± 3.146
Organic Matter (%)	0.990 ± 0.027	1.168 ± 0.086
soil water content (% g/gdried soil)	8.780 ± 0.944	14.570 ± 0.755
pH (H_2O)	6.320 ± 0.096	6.765 ± 0.473
EC (dS m^{-1})	0.075 ± 0.011	0.073 ± 0.012
Carbonates (% $CaCO_3$)	0.697 ± 0.094	0.585 ± 0.399
F^- (mg kg^{-1})	0.000 ± 0.000	1.235 ± 1.235
Cl^- (mg kg^{-1})	11.292 ±2.183	10.815 ± 1.262
NO_2^- (mg kg^{-1})	0.349 ±0.124	0.442 ± 0.048
NO_3^- (mg kg^{-1})	123.821 ± 20.666	59.649 ± 13.204
PO_4^{3-} (mg kg^{-1})	1.134 ± 0.248	3.081 ± 1.928
SO_4^{2-} (mg kg^{-1})	12.481 ± 0.413	8.727 ± 1.083
K^+ (mg kg^{-1})	6.373 ± 0.953	6.408 ± 1.629
Na^+ (mg kg^{-1})	11.262 ± 1.839	7.856 ± 0.892
Ca^{2+} (mg kg^{-1})	1324.575 ± 1266.033	63.584 ± 18.549
Mg^{2+} (mg kg^{-1})	16.622 ± 13.668	3.598 ± 1.302

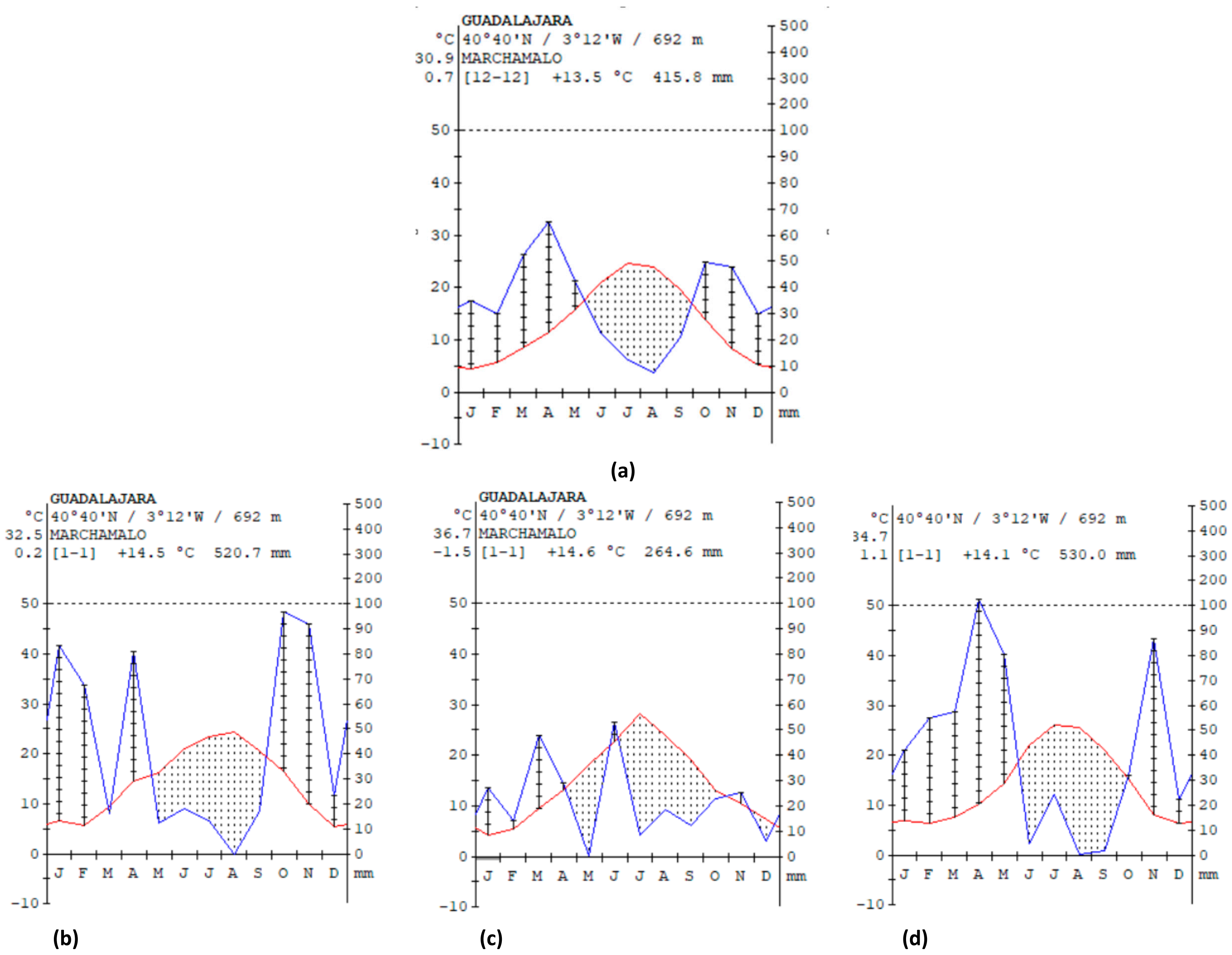

Figure S1 Walter-Lieth diagrams for 2010-2021 period (a), 2014 (b), 2015 (c) and 2016 (d) from Marchamalo meteorological station (40º 40′ N 3º 12′W).

mean mensual temperature (ºC); mensual precipitation (mm)
